# Supplementary figures and images for: Self-referencing rates in biological disciplines
Source: Front Res Metr Anal. 2023 Sep 22;8:1215401. doi: 10.3389/frma.2023.1215401 (PMC10556682; doi:10.3389/frma.2023.1215401)

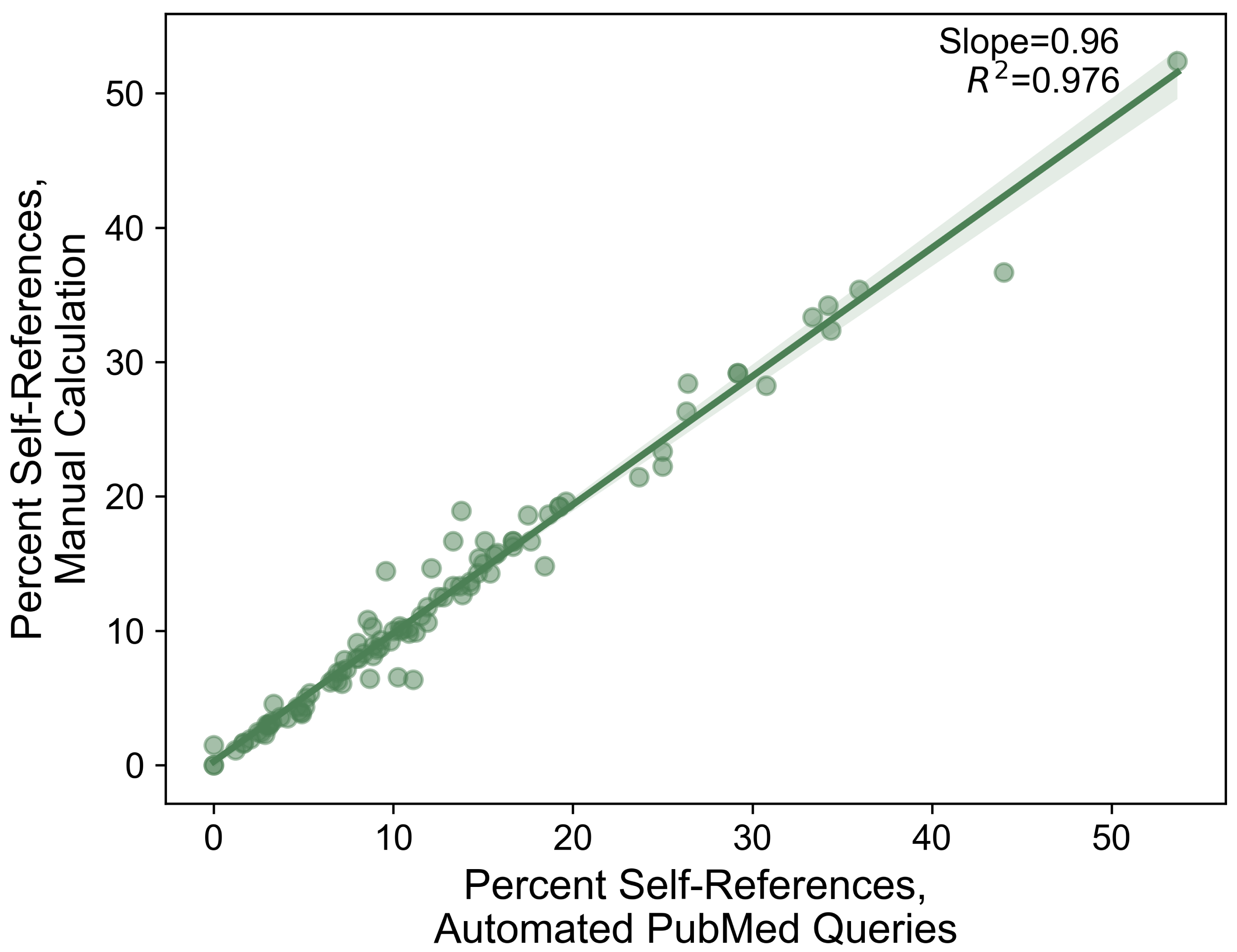

Supplement: Supplementary Figure S1 — Comparison of manual vs. automated calculation of self-referencing rates. A total of 100 publications were randomly selected from all publications evaluated. Self-referencing rates were calculated manually by examining the reference list in each publication, then compared to the self-referencing rate calculated via the automated PubMed query pipeline. [file Image_1.TIF]

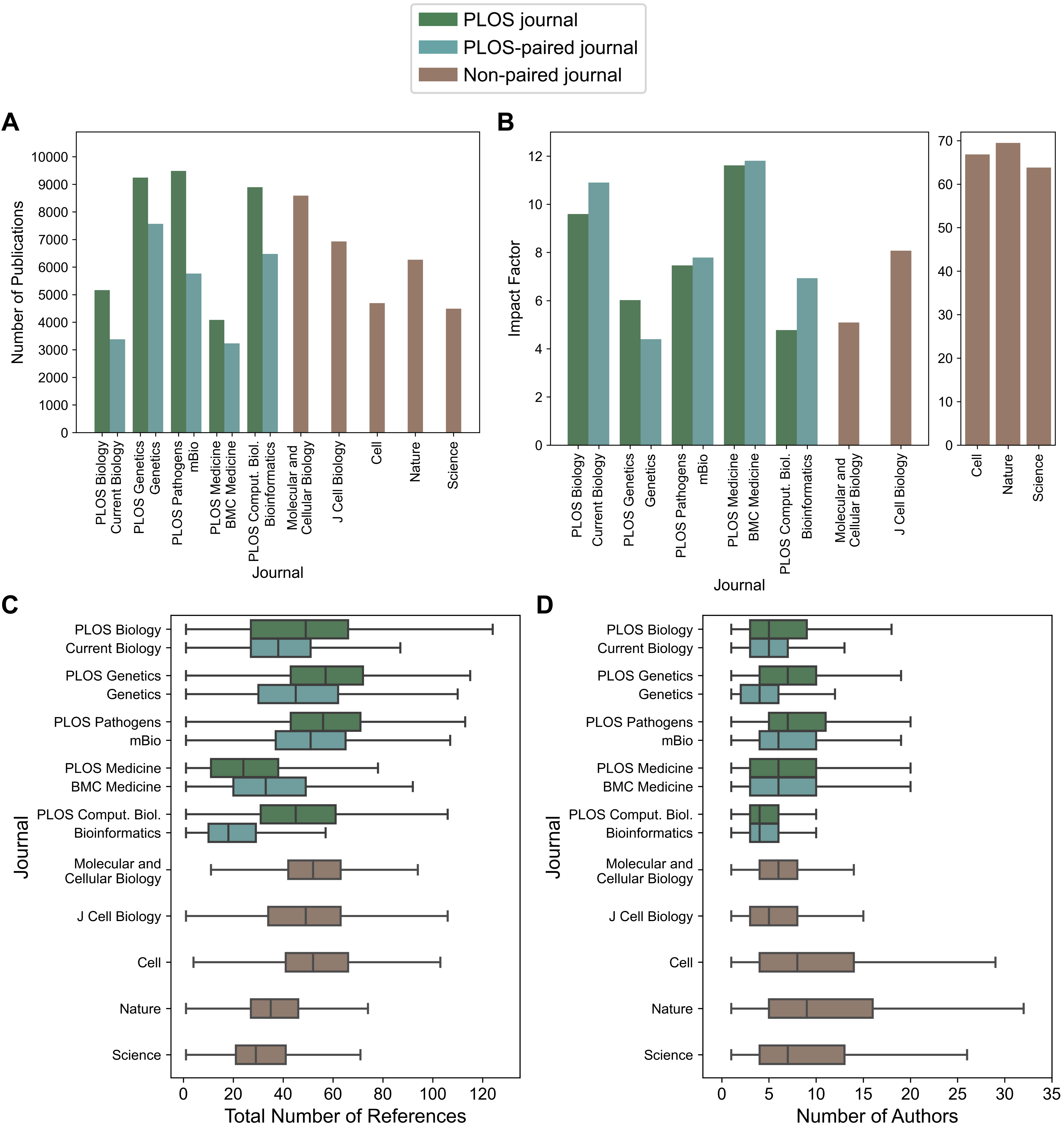

Supplement: Supplementary Figure S2 — Comparison of number of publications, impact factor, number of references, and number of authors across journals. (A) Total number of publications evaluated in this study for each journal. (B) Impact factor of each journal. (C) Distributions of total number of references within the primary publications evaluated for each journal. (D) Distributions of the number of authors for the primary publications evaluated for each journal. [file Image_2.TIF]

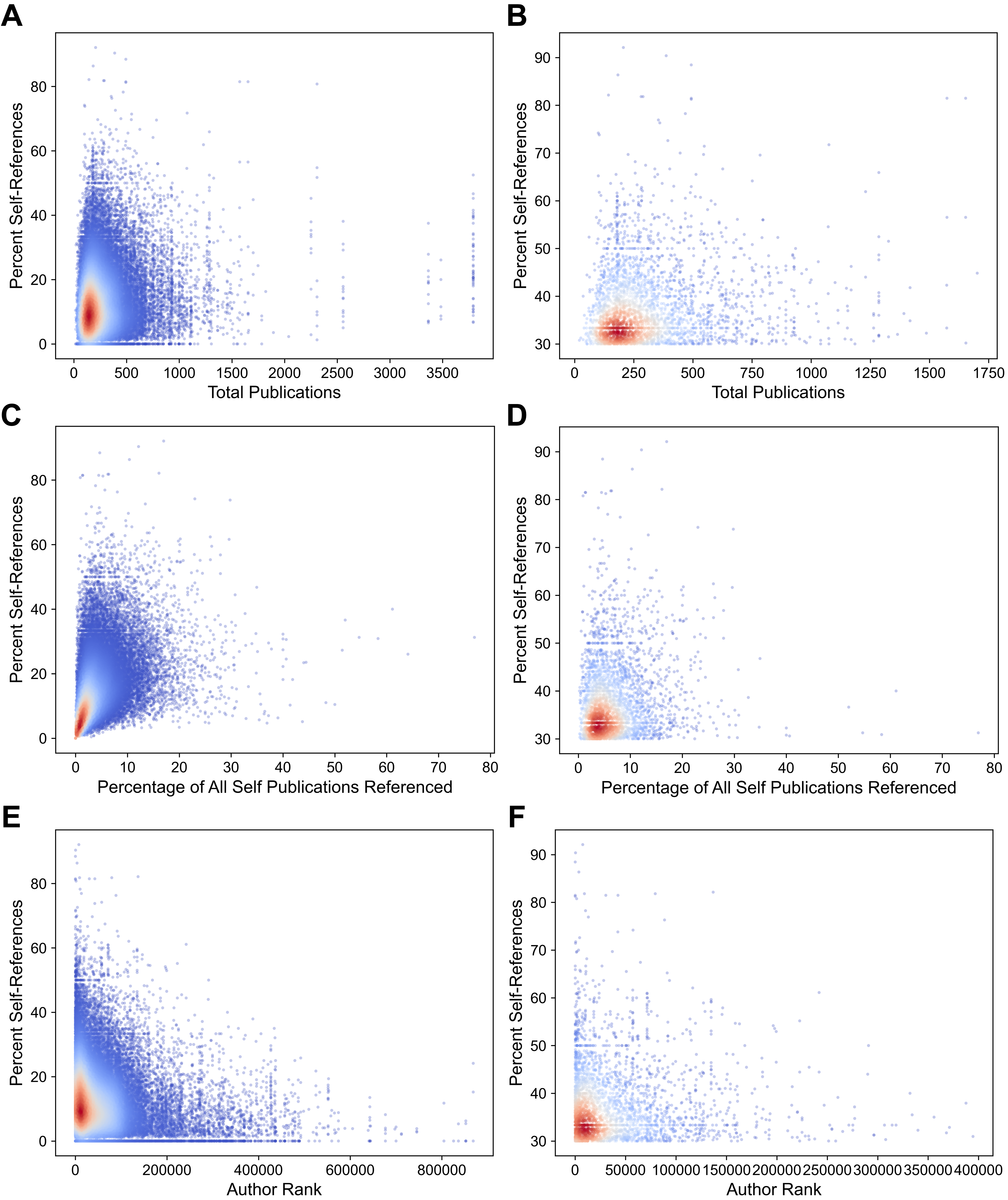

Supplement: Supplementary Figure S3 — Self-referencing rates as a function of total publications, fraction of total publications referenced, and author rank. (A) Scatterplot depicting the percentage of self-references vs. total publications. (B) Scatterplot focusing on the percentage of self-references vs. total publications only for publications with very high self-referencing rates (≥30%) and authors with fewer than 2,000 total publications. (C, D) Similar to (A, B), but with the percentage of total publications self-referenced serving as the x-axis variable. (E, F) Similar to (A, B), but with author rank (Ioannidis et al., 2019, 2020) serving as the x-axis variable. For all panels, only the subset of authors appearing in the ranked-author database were included in analyses. Author rank in (E, F) was limited to 1 million and 400 k, respectively, for visual clarity only. Point density was estimated using a kernel density estimate and colored accordingly, with dark red indicating high-density regions and dark blue indicating low-density regions. [file Image_3.TIF]

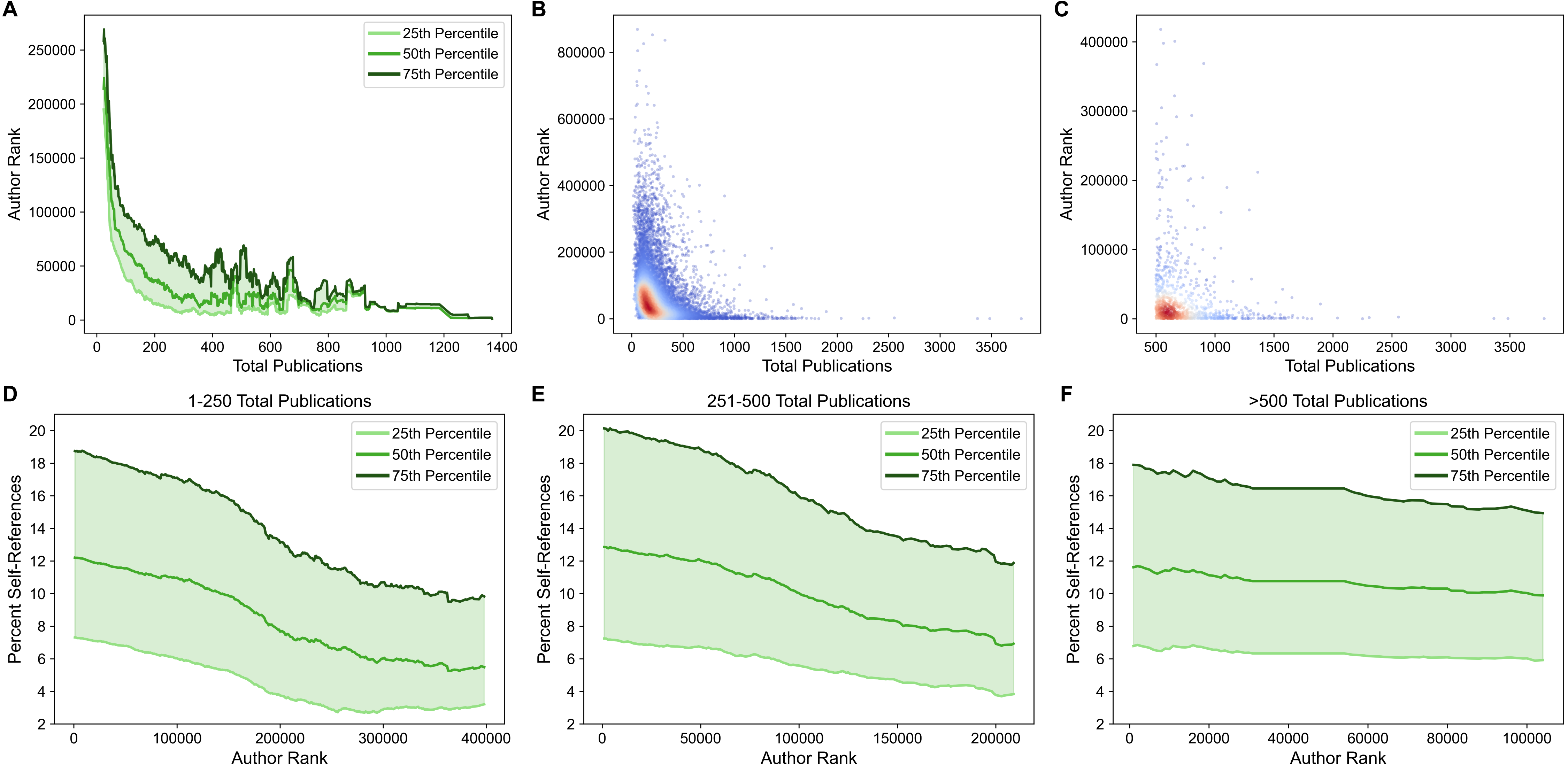

Supplement: Supplementary Figure S4 — Author rank vs. total publications. (A) Plot depicting shifts in author-rank quartile values as a function of total publications. (B) Scatterplot depicting author rank as a function of total publications. (C) Scatterplot of author rank vs. total publications, focusing on authors with ≥500 total publications. Authors with an unusually large number of total publications are concentrated among top-ranking authors. For (B, C), point density was estimated using a kernel density estimate and colored accordingly, with dark red indicating high-density regions and dark blue indicating low-density regions. (D–F) Shifts in percent self-reference distributions as a function of author rank, grouped by total publications as indicated in the title of each panel. [file Image_4.TIF]
